# Diagnosis prediction of tumours of unknown origin using ImmunoGenius, a machine learning-based expert system for immunohistochemistry profile interpretation

**DOI:** 10.1186/s13000-021-01081-8

**Published:** 2021-03-11

**Authors:** Yosep Chong, Nishant Thakur, Ji Young Lee, Gyoyeon Hwang, Myungjin Choi, Yejin Kim, Hwanjo Yu, Mee Yon Cho

**Affiliations:** 1 Department of Hospital Pathology, College of Medicine, The Catholic University of Korea, 271, Cheonbo-ro, Uijeongbu, 11765 Gyeonggi-do Republic of Korea; 2 Postech-Catholic Biomedical Engineering institute, College of Medicine, The Catholic University of Korea, Seoul, Republic of Korea; 3 Dasom X, Inc., Seoul, Republic of Korea; 4 Department of Creative Information Technology, POSTECH, Pohang, Republic of Korea; 5 University of Texas Health Science Center, Houston, TX USA; 6 Computer Science and Engineering, POSTECH, Pohang, Republic of Korea; 7 Department of Pathology, Yonsei University, Wonju College of Medicine, Wonju, Republic of Korea

**Keywords:** Database, Expert system, Machine learning, Immunohistochemistry, Probabilistic decision tree

## Abstract

**Background:**

Immunohistochemistry (IHC) remains the gold standard for the diagnosis of pathological diseases. This technique has been supporting pathologists in making precise decisions regarding differential diagnosis and subtyping, and in creating personalized treatment plans. However, the interpretation of IHC results presents challenges in complicated cases. Furthermore, rapidly increasing amounts of IHC data are making it even harder for pathologists to reach to definitive conclusions.

**Methods:**

We developed ImmunoGenius, a machine-learning-based expert system for the pathologist, to support the diagnosis of tumors of unknown origin. Based on Bayesian theorem, the most probable diagnoses can be drawn by calculating the probabilities of the IHC results in each disease. We prepared IHC profile data of 584 antibodies in 2009 neoplasms based on the relevant textbooks. We developed the reactive native mobile application for iOS and Android platform that can provide 10 most possible differential diagnoses based on the IHC input.

**Results:**

We trained the software using 562 real case data, validated it with 382 case data, tested it with 164 case data and compared the precision hit rate. Precision hit rate was 78.5, 78.0 and 89.0% in training, validation and test dataset respectively. Which showed no significant difference. The main reason for discordant precision was lack of disease-specific IHC markers and overlapping IHC profiles observed in similar diseases.

**Conclusion:**

The results of this study showed a potential that the machine-learning algorithm based expert system can support the pathologic diagnosis by providing second opinion on IHC interpretation based on IHC database. Incorporation with contextual data including the clinical and histological findings might be required to elaborate the system in the future.

**Supplementary Information:**

The online version contains supplementary material available at 10.1186/s13000-021-01081-8.

## Background

Immunohistochemical staining (IHC) is an essential staining method for differentiating tumor origin in pathologic diagnosis. It enables to infer the origin of cells by investigating the expression of specific antigens in the tissue [1–6]. In 1941, Dr. Albert Coons developed an indirect form of immunofluorescence staining technique [1, 7]. Initially, it was designed for staining fresh tissue samples and samples were visualized by fluorescence microscopy. However, with the introduction of enzyme-conjugated antibodies and paraffin-embedding, IHC became a regularly used assay in the diagnosis of pathological conditions [2–6]. Simultaneously, the role of IHC has been extended from classifying the cellular origin of tumours to the subtyping tumours, determining treatment efficacy, predicting patient prognosis (prognostic marker), and finally differentiating precancerous lesions by evaluating the molecular changes [1–3, 8].

However, the rapidly expanding knowledge about IHC positivity in each neoplasm often leads to conflicting interpretations in routine practices, especially in some complicated cases [9]. For example, a combination of TTF-1 (lung and thyroid), galectin-3 (100% in papillary thyroid cancers), and napsin A (lung adenocarcinomas) is used to determine the tumour origin of a lung mass in patients with thyroid nodules [10, 11]. However, in different lung cancer subtypes, TTF-1 positivity changes from 21 to 91%, and galectin-3 shows 49% positivity in the subset of lung adenocarcinomas, and napsin A shows a positivity of less than 5% in thyroid cancers, which means that the IHC results by themselves cannot exclude the rare exceptions [11–13]. The interpretation of IHC results can be biased depending on the experience and knowledge of the individual pathologists [2, 4, 6]. Presently, thousands of new antibodies and IHC staining data from various tumours are available to researchers. Over a hundred thousand studies using IHC-based assays have been published since 2000. Therefore, it is not feasible for the pathologists to memorize the expression of all the molecular markers recognized by the constantly evolving repertoire of antibodies in tumours from different tissues of origin [14].

Algorithmic approaches and standardized IHC panels for certain diagnoses have been used to solve this problem [9, 14, 15]. However, in clinical practice, each case is unique and sensitive, and generalized application of particular IHC panels in some cases can be time-consuming and labour-intensive.

Thus, we developed an expert system using computer software, in the form of an iOS and Android mobile application-based on a machine-learning algorithm and IHC database IHC that assists pathologists in making a precise diagnosis.

## Methods

This study was approved by the Institutional Review Board of the Catholic University of Korea, College of Medicine (SC17RCDI0074).

### Development of machine-learning algorithm using probabilistic decision tree

Bayesian theorem is one of the main topic in the field of probability theory and statistics. This indicates a relationship for random variables between conditional probabilities and marginal probabilities. According to Bayesian theorem, the post-event probability can be calculated when the pre-event probability is given. Bayes’ theorem is stated mathematically as P(B) ≠ 0, where A and B are events [16]. P(A|B) and P(B|A) are the conditional probabilities, such that the likelihood of event A occurring, given that B has occurred and vice versa, respectively. P(A) and P(B) are the probabilities of observing A and B independently of each other [16].

$$ P\left(A\left|B\right.\right)=\frac{P\Big(B\left|A\Big)\right.P(A)}{P(B)} $$

**Fig. 1:**
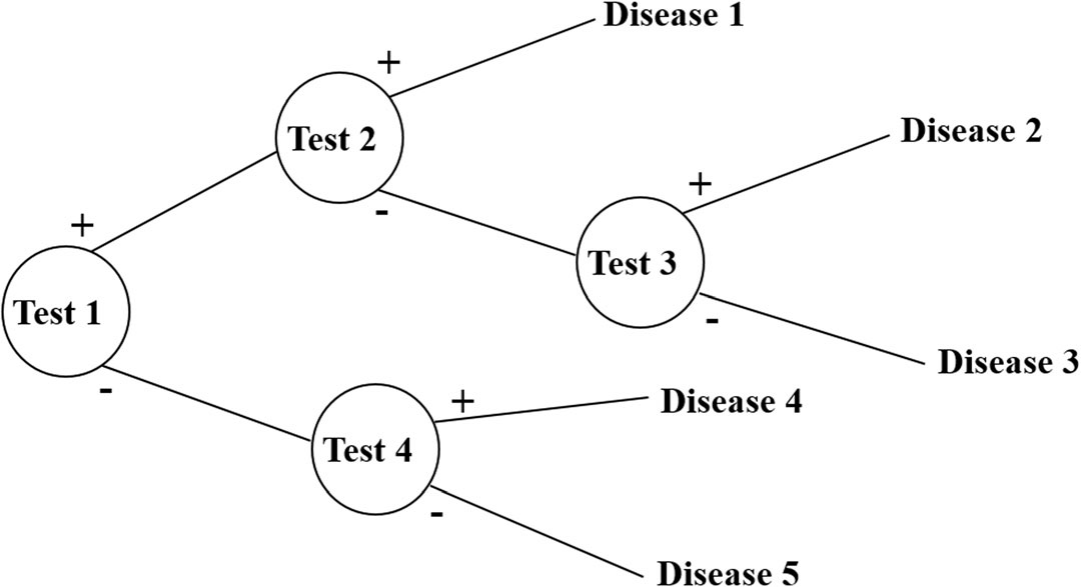
Probabilistic decision tree for the machine-learning algorithm in diagnostic tests and disease

IHC results are binary and the probability of positive and negative IHC in each neoplasm is empirically known by pathologists and relatively well documented in the textbooks and literature (Fig. 1). Although incidence of each neoplasm should be pre-event probability, incidence of each neoplasm varies with various other factors such as ethnicity, and we are dealing with the hypothetical probability that is only based on IHC results. In addition, the effect of pre-event probability can be too high when additional conditions are few and its effect can be low when additional conditions are many enough. Therefore, we hypothetically supposed that the pre-event probability is neglectable for computation.

Collectively, we need a database of a 2 × 2 table with tests, diseases, and the probability of positivity of each test for each disease. Test results obtained are binary. The probability of positivity signifies the number of positive cases among all the cases of the disease. Once the test results are obtained, the probability for each disease can be calculated by multiplying the prior probability and the probabilities of each test being positive or negative, to indicate the illness with the highest probability, by comparing post probability.

**Fig. 2:**
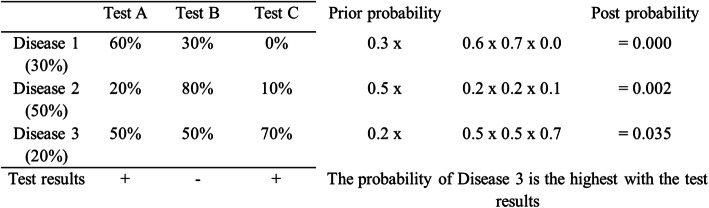
The prior and post probability based on Bayes’ theorem

Let us take an example as in Fig. 2. Suppose that the pre-event disease probability is 30% for Disease 1, 50% for Disease 2, and 20% for Disease 3, and the known probability of positive results of Test A, B, and C for each disease is as shown in the table of Fig. 2. If we get the results of Test A, B, C as positive, negative, and positive, we can calculate post probability as the equations next to the table. As a result, the probability of Disease 3 is the highest upon the test results.

### Construction of IHC database

As shown in Supplementary table 1, important textbooks on IHC such as Classification of Tumours Series (IARC, Lyon, France) and literature from World Health Organization (WHO), were used to build an IHC database based on the IHC expression profile of all tumours [4, 5, 17–28]. Over 5000 different neoplasms were recorded based on the WHO classification. Neoplasms without IHC expression profile were excluded. Differences in the IHC profile of tumour subtypes, were recorded separately from the primary type.

Each tumours IHC positivity was recorded as showed in the textbook. If there was no exact numerical value attributed to the positivity, arbitrary expressions such as “always positive”, “often positive”, or “rarely/ occasionally positive” were assigned. The positivity of each tumour was described as: “always”: 95%; “often”: 75%; “in about a half of cases”: 50%; “seldom”: 30%; “rarely/ occasionally”: 10%; and “never”: 0%. If the positivity differs between textbooks, the average value was used in the database. IHC database showed in Supplementary Fig. 1.

Around 600 antibody names and their synonyms used in IHC were recorded using the textbooks and reviewed with the online references Supplementary table 2.

### Development of ImmunoGenius, a mobile application for iOS and android

**Fig. 3:**
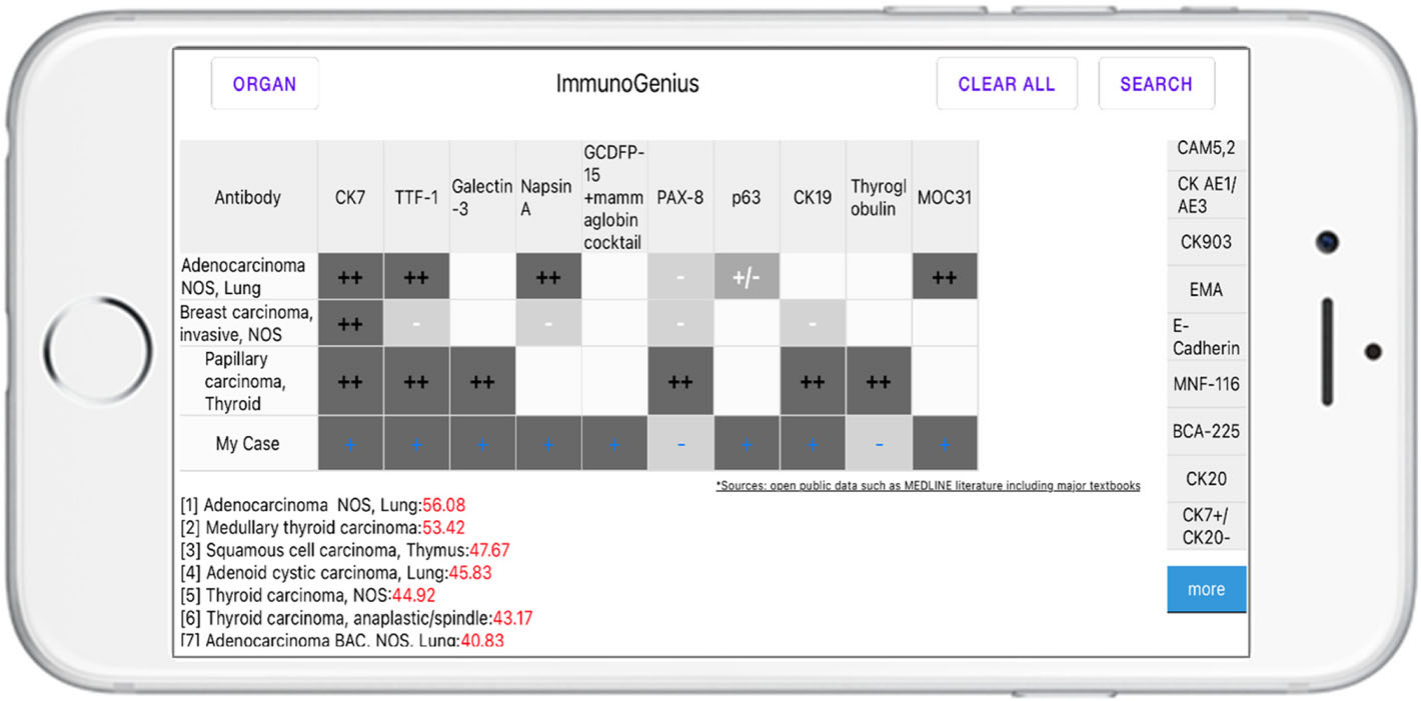
The screenshot of the mobile application “ImmunoGenius”

**Fig. 4:**
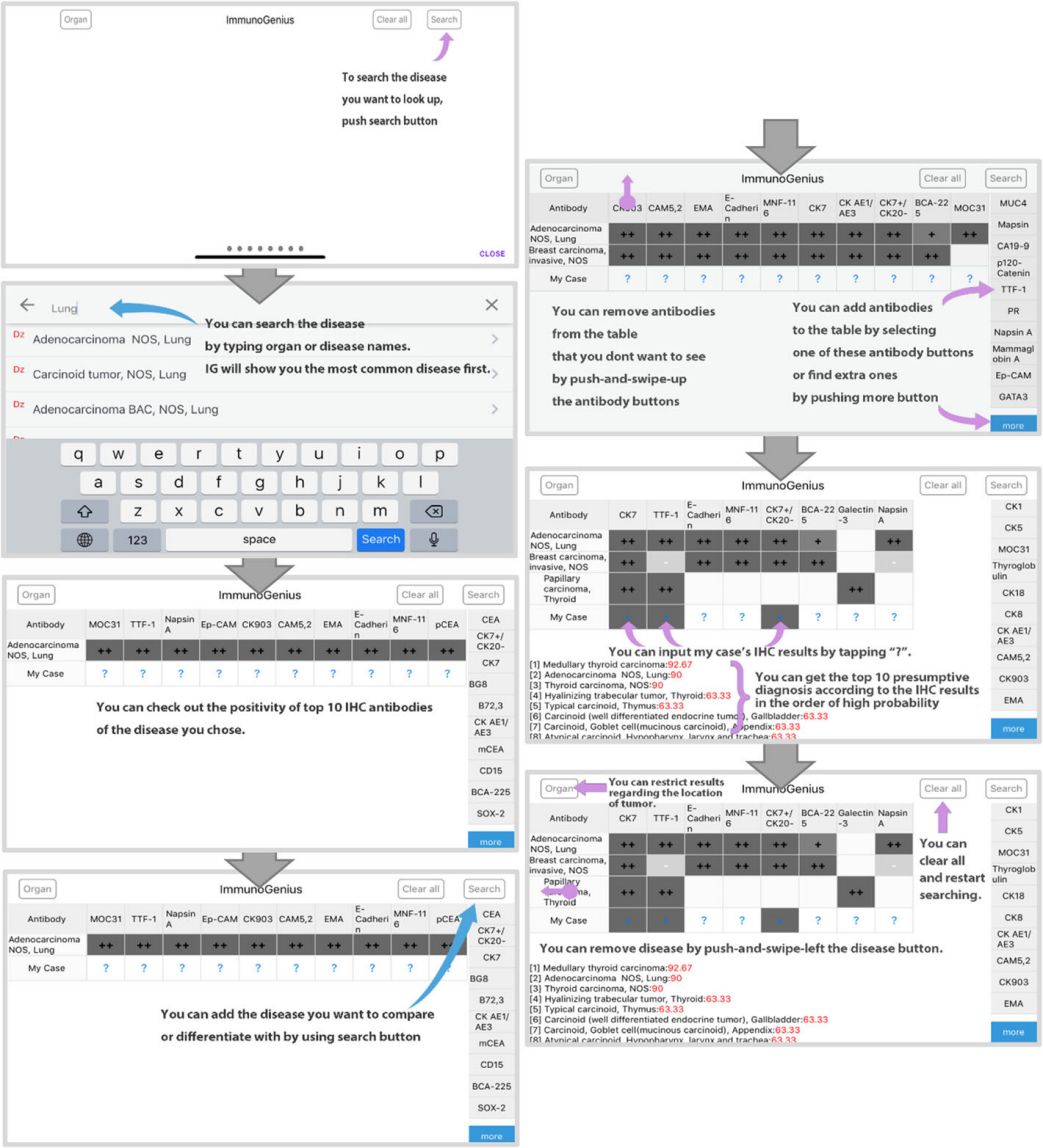
Exemplary flowchart for ImmunoGenius

The “ImmunoGenius” mobile application for iOS and Android was developed using NoSQL (Fig. 3) and can be accessed on iPhones, Android phones, and iPads. It is designed to search for diseases and upon selection of the illness it generates a table with the IHC antibody names in the first row and disease name in the left column. The IHC profiles are showed in the corresponding cells designated as “++” for 75–100% positivity, “+” for 50–74%, “+/−” for 30–49%, “−/+” for 10–29%, and “–” for 0–9% shown with graded shades (Fig. 4). Individuals can compare the different IHC profiles and add or remove the diseases and IHC antibodies to customize the table. Importantly, individuals can add their IHC results through a button on the right-hand side. Once the IHC results are inserted, the diagnosis presumption algorithm calculates the top 10 most probable diagnoses, which are shown along with the estimated probability (red numbers). The detailed user instructions and software download is available at homepage: https://immunogenius.wixsite.com/website)

google play store: https://play.google.com/store/apps/details?id=com.dasomx.ig&hl=ko

you tube video:  https://youtu.be/0EUQKCmAXc8

### Validation of diagnosis presumption algorithm using patient data

To prove the precision of the diagnosis presumption algorithm, IHC profile data was generated for specific cases and diagnosed by pathologists using conventional methods. These were then compared with the top 10 results from the presumptive diagnoses algorithm. The IHC profile data of 1000 tumours of unknown origin (TUOs) collected between 2010 to 2017 from the Yeouido and Seoul St. Mary’s Hospital, College of Medicine, The Catholic University of Korea were used in this study. Any data related to patient identification, except the original diagnosis and the IHC results, were blinded before data processing. In addition, we collected the IHC profile data of 164 TUOs for test dataset diagnosed in 2020 from the archives at Uijeongbu St. Mary’s Hospital, College of Medicine, The Catholic University of Korea. TUOs were defined as the cases in clinical or pathological situation, where the immunohistochemical differential diagnosis is needed to differentiate between primary or metastatic lesions, or between variable subtypes of cancers, for confirmative diagnosis. In such cases, the histological findings alone cannot exclude the possibility of misdiagnosis or misclassification (e.g. determination of tumour origin in ascites, pleural fluid, or lymph nodes; determination of primary or metastatic lesions and pathologic subtyping in the needle biopsy samples of lung, liver, or kidney, where metastasis is common and clinicoradiologic findings are not confirmative). For training and validation, the retrieved database was divided into 6:4. The cases with inadequate IHC profiles such as the absence of markers for tumour origins, IHC less than three antibodies, inconclusive results were excluded. However, only prognostic markers such as EGFR or p53 were eliminated. Supplementary Fig. 2 showed an example of retrieved IHC profile dataset from patients. The precision of diagnosis presumption algorithm was confirmed by the inclusion of the diagnosis obtained by conventional methods in the top 10 presumptive diagnoses generated by the algorithm. It is considered to be inclusive, without significant difference in the IHC profile, between the initial and presumptive diagnosis, but the only difference in location (e.g., gastrointestinal stromal tumour of the stomach vs. small intestine). The hit rate of training and validation data was compared to prove the functionality of the algorithm. The algorithm is considered validated, if there is no statistically significant difference between the training and validation dataset. After training and validation, algorithm was tested with dataset of another institute (external validation).

### Statistical analysis

Time and computer complexity were accessed by testing the mobile application. Chi-square test was used to compare the hit rate between original and presumptive diagnoses. A web-based statistical analysis (“http://web-r.org”) was used for statistical analysis.

## Results

### Construction of IHC database. Recruitment of training, validation, and test dataset

The detailed information related to 2009 different types of cancer, 584 IHC antibodies, and their IHC profiles were recorded in the IHC database. Five hundred sixty-two cases were used for the training dataset, 382 cases were used for the validation dataset and 164 cases for test dataset.

### Training data

The recruited training and validation data of the tumours were from 562 and 382 cases, respectively. On an average, 6.8 IHC antibodies (ranged 1–13) were used for diagnosis. A wide variety of tumours from 32 organs were included. The organ and the original diagnoses of the training data cases are shown in Tables 1 and 2. The common organs were lung (20.6%), liver (9.8%), kidney (6.6%), stomach (6.6%), and large intestine/rectum (5.3%) (Table 1). Ascites and peritoneum consist of 5.7%, while pleural fluid and pleura comprised of 5.2% (Table 1) of the cases. Primary carcinoma consists of 41.3% of the cases, followed by metastatic carcinoma (26.9%), benign mesenchymal tumour (21.4%), mild (normal) lesion (5.9%), and malignant mesenchymal tumour (4.6%) (Table 2). The hit rate of the presumptive diagnosis of the training data (top 10) was 78.5% (Table 3). The error rates being the highest at 30.8% in malignant mesenchymal tumours, followed by metastatic carcinoma (25.8%), benign mesenchymal tumours (23.3%), primary carcinoma (18.1%), and benign (normal) lesion (12.1%).

**Table 1:** The organs of the training and validation dataset of TUO

Organ	Training data		Validation data		Test data	
	No.	%	No.	%	No	%
Ascites (cell block)	19	3.4%	11	2.9%	1	0.6%
Pleural fluid (cell block)	15	2.7%	12	3.1%	0	0.0%
Lymph node	12	2.1%	9	2.4%	8	4.9%
Peritoneum	13	2.3%	8	2.1%	2	1.2%
Pleura	14	2.5%	7	1.8%	2	1.2%
Lung	**116**	**20.6%**	**75**	**19.6%**	**26**	**15.9**%
Liver	**55**	**9.8%**	**43**	**11.3%**	**33**	**20.1**%
Kidney	**37**	**6.6%**	**31**	**8.1%**	**15**	**9.1**%
Breast	17	3.0%	11	2.9%	1	0.6%
Soft tissue	38	6.8%	14	3.7%	6	3.6%
Female genital tract including uterus and vulva, vagina	17	3.0%	12	3.1%	**18**	**10.1**
Adnexa	8	1.4%	6	1.6%	0	0.0%
Bladder and urinary tract	7	1.2%	5	1.3%	1	0.6%
Adrenal gland	10	1.8%	5	1.3%	1	0.6%
Prostate	15	2.7%	11	2.9%	0	0.0%
Testis	8	1.4%	5	1.3%	0	0.0%
Pancreas	7	1.2%	4	1.0%	3	1.8%
Stomach	**37**	**6.6%**	**20**	**5.2%**	**9**	**5.5**%
Small intestine	4	0.7%	1	0.3%	5	3.0%
Large intestine and rectum	**30**	**5.3%**	**23**	**6.0%**	**12**	**7.3**%
Gallbladder	3	0.5%	1	0.3%	0	0.0%
Appendix	**2**	0.4%	1	0.3%	0	0.0%
Brain (CNS)	11	2.0%	6	1.6%	**14**	**8.5%**
Meninges	22	3.9%	16	4.2%	0	0.0%
Naso- and oropharynx	13	2.3%	10	2.6%	2	1.2%
Skin	14	2.5%	11	2.9%	1	0.6%
Bone	6	1.1%	5	1.3%	3	1.8%
Thyroid gland	2	0.4%	1	0.3%	0	0.0%
Thymus	3	0.5%	2	0.5%	0	0.0%
Salivary gland	**5**	0.9%	3	0.8%	0	0.0%
Eye	2	0.4%	0	0.0%	2	1.2%
Total	**562**	**100.0%**	**382**	**100.0%**	**164**	**100.0%**

Abbreviations: *TUO* Tumor of unknown origin

**Table 2:** The original diagnoses of the training and validation dataset of TUO

Organ	Training data				Validation data				Test data			
	No.	%	Errors	%	No.	%	Errors	%	No.	%	Errors	%
Primary carcinoma	232	41.3%	42	18.1%	163	42.7%	25	15.3%	89	54.3%	7	7.9%
Metastatic carcinoma	151	26.9%	39	25.8%	98	25.7%	26	26.5%	19	11.6%	4	21.1%
Benign (normal) lesion	33	5.9%	4	12.1%	22	5.8%	3	13.6%	13	7.9%	1	7.7%
Benign mesenchymal tumor	120	21.4%	28	23.3%	80	20.9%	24	30.0%	24	14.6	4	16.7%
Malignant mesenchymal tumor	26	4.6%	8	30.8%	19	5.0%	6	31.6%	19	11.6%	2	10.5%
	562	100.0%	121	21.5%	382	100.0%	84	22.0%	164	100%	18	11%

Abbreviations: *TUO* Tumor of unknown origin

**Table 3:** The comparison of Precision error rates between the training and validation dataset of TUO

Precision diagnosis	Training data		Validation data		Test data		Total	
	No.	%	No.	%	No.	%	No.	%
Accurate results	441	78.5%	298	78.0%	146	89%	885	79.9%
Error results	121	21.5%	84	22.0%	18	11%	223	20.1%
Total	562	100.0%	382	100.0%	164	100%	1108	100.0%

Abbreviations: *TUO* Tumor of unknown origin

### Validation data

The organs and the original diagnoses are shown in Tables 1 and 2. The common organs in the validation dataset were similar to the training dataset, which are lung (19.6%), liver (11.3%), kidney (8.1%), stomach (5.2%), and large intestine/rectum (6.0%) (Table 1). Ascites and peritoneum consist of 5.0%, while pleural fluid and pleura comprised of 4.9% of the cases (Table 1). Primary carcinoma consists of 42.7% of the cases, followed by metastatic carcinoma (25.7%), benign mesenchymal tumour (20.9%), benign (normal) lesion (5.8%), and malignant mesenchymal tumour (5.0%). The hit rate of the presumptive diagnosis of the validation data (top 10) was 78.0% (Table 3), with the highest error rates at 31.6% in malignant mesenchymal tumours, followed by benign mesenchymal tumours (30.0%), metastatic carcinoma (26.5%), primary carcinoma (15.3%) and benign (normal) lesion (13.6%).

### Test data

We exploited 164 patients’ cases for the test dataset. The organ and the original diagnoses are shown in Tables 1 and 2. The most common organs were lung (15.9%), liver (20.1%), female genital tract including uterus and vulva, vagina (10.1%), kidney (9.1%), brain (8.5%), large intestine and rectum (7.3%) and stomach (5.5%) (Table 1). Primary carcinoma consists of 54.3% of the cases, followed by metastatic carcinoma (11.6%), benign (normal) lesion (7.9%), benign mesenchymal tumour (14.6%), and malignant mesenchymal tumour (11.6%) (Table 2). The hit rate of the presumptive diagnosis of the training data (top 10) was 89% (Table 3). The error rates being the highest at 21.1% in metastatic carcinoma, followed by benign mesenchymal tumours (16.7%), malignant mesenchymal tumours (10.5%), primary carcinoma (7.9%), and benign (normal) lesion (7.7%).

### The precision error rates between training, validation, and test dataset

The error rates of the precision diagnosis were 21.5 and 22.0% for training and validation datasets, respectively (Table 3); which was not significantly different (*p*-value = 0.866). The error rates of the precision diagnosis for test dataset was much less up to 11.0%. The overall hit rate was 79.9% (Table 3).

### Example of application

Let us take an example application of ImmunoGenius in real pathology practice. Recently we experienced a 50-year-old woman with a 1.5 cm-sized lung mass in her left upper lobe. She had a history of lumpectomy due to invasive ductal carcinoma 5 years ago. In addition, a 1.5 cm-sized thyroid nodule was found during the assessment. Based on this clinical information, we could hypothesize that this nodule can be primary lung adenocarcinoma, recurrent invasive ductal carcinoma, or metastatic thyroid papillary carcinoma. On H & E staining of needle biopsied sample, the tumor was adenocarcinoma with acinar and papillary pattern and irregular nuclei with frequent indistinctive nucleoli, which can be adenocarcinoma of either primary pulmonary, secondary mammary, and secondary thyroidal origin. In this practical setting, most pathologists would choose to perform IHC for CK7, CK20, TTF-1, GCDFP-15, galectin3, and napsin A for the differential diagnosis. We performed these markers at the first round of IHC and it was positive for CK7, TTF-1, galectin 3 and napsin A, and negative for CK20 and GCDFP-15 (Supplementary Fig. 3) TTF-1 and napsin A are very important markers for the lung cancer diagnosis, GCDFP-15 is important for breast cancer, and CK7, galectin 3, and TTF-1 are important for thyroid cancer diagnosis. However, as we see in the textbook table, napsin A can be also found in 5% of thyroid cancers as well as galectin 3 can be found up to 50% of lung adenocarcinoma. Therefore, we can rule out the possibility of breast cancer, but it can either be lung or thyroid carcinoma. So we had to get help from ImmunoGenius application on this case to check the real probabilistic difference calculated by these IHC profiles and the probability of both adenocarcinoma and thyroid carcinoma turned out to be similar as 64% (Supplementary Fig. 3). For the confirmative diagnosis, we additionally performed the IHC for CK19, thyroglobulin, MOC31, PAX8 and p63. As a result, we could find the most probable diagnosis is lung cancer with 56% probability and thyroid carcinoma showed 53% of probability when it is an anaplastic histologic variant (Supplementary Fig. 3). With these results, we could rule out thyroid carcinoma more confidently with presumptive diagnosis prediction by ImmunoGenius.

## Discussion

In the present study, we verified the estimated the diagnostic probability of certain TUOs, using IHC results, by probabilistic decision tree and corresponding mobile application. The precision diagnosis drawn by the probabilistic decision tree algorithm, at the hit rate of 79.9%, can be a convincing assistant in decision making for pathologists. The hit rate rates between training, validation dataset were not statistically significant (78.5% vs. 78.0%, *p*-value = 0.866).

The hit rate of the presumptive diagnosis was generally poor compared to the results of our prior validation study using lymphoma cases that showed 95% precision hit rate [29]. It is mainly due to the magnitude of the disease entities (2009 vs. 104). The common organs in the data used were lung, liver, kidney, ascites and peritoneum, and pleural fluid/pleura where metastatic lesions are often found in clinical practice. In case of the lungs, IHC was commonly used for subtyping between small cell, adeno, and squamous cell carcinoma, as well as determining the origin of the tumour, and whether it is primary or metastatic. In case of the kidneys, IHC was also used for subtyping between clear cell, chromophobe, papillary, etc., as well as determining whether it is primary or metastatic. For ascites and peritoneum, IHC was used for determining whether it is a metastatic carcinoma, or reactive mesothelial cells/macrophages. Moreover, in case of pleural fluid and pleura, IHC was used for determining whether it is metastatic adenocarcinoma (from the lung), mesothelioma, or reactive mesothelial cells/macrophages. Furthermore, in case of stomach, the primary differential diagnosis was between spindle cell neoplasms including gastrointestinal stromal tumours (GIST), schwannoma, and leiomyoma. Finally, in case of colon/rectum, benign spindle cell neoplasms and neuroendocrine cell tumours (carcinoid) were the most common disease.

The primary cause of inaccurate presumptive diagnosis was atypical IHC profiles (compared to that described in the textbook; about two thirds). The major causes of inaccurate presumptive diagnosis included overlapping IHC profiles between adenocarcinomas of the gastrointestinal tract, the origin of squamous cell carcinoma (no site-specific marker for squamous cell carcinoma), mesenchymal neoplasia that express both epithelial and mesenchymal markers, tumours with mixed or combined entities (e.g. squamous transformation of adenocarcinoma of the lung after chemotherapy, combined germ cell tumour, etc.), and tumours with no disease-specific markers. The cases with typical IHC markers tended to show accurate presumptive diagnosis. In other words, the precise differential diagnosis cannot be made only using the IHC profile in about 22% of the cases, and clinicopathologic findings along with the patient history should be considered. Thus, this algorithm should be used and interpreted with contextual information in a comprehensive and integrated manner. This study clearly showed the feasibility and clinical utility of making a diagnosis using the probabilistic decision tree algorithm and iOS and Android mobile application in the differential diagnosis of the tumours using IHC profiles.

## Conclusions

The overall hit rate of this machine-learning algorithm was 79.9%, and the hit rate rates were not significantly different between training and validation data, and it was much lower in test data, thus showing a relatively robust generalization. Disease-specific markers, overlapping IHC profiles between diseases, a lack of site-specific markers, mixed/combined tumours, and atypical IHC profile are the leading causes of error in this system. However, this system will be useful to assist the pathologists in making precise decisions during the disease diagnosis Integrated interpretation with contextual information such as clinical and pathological findings should be considered, along with the use of this application, before making a final decision. Further studies for recommending IHC panels for particularly complex problems regarding differential diagnosis and application of artificial neural network algorithms to optimize the disease diagnosis [30, 31], organ incidence, and antibody weight are needed in the future.

## Supplementary Information

**Additional file 1: Figure S1.** The example of the IHC database.

**Additional file 2: Figure S2.** The example of the patients IHC profile dataset for training and validation of the diagnosis presumption algorithm.

**Additional file 3: Figure S3.** The example of application using a case of tumor of unknown origin.

**Additional file 4: Table S1.** The reference books used for IHC database build. **Table S2.** Online references used for IHC antibody name documentation.

## Data Availability

The datasets used and/or analyzed during the current study are available from the corresponding author on reasonable request.

## Abbreviations

IHC: Immunohistochemistry
WHO: World Health Organization
TUOs: Tumours of unknown origin
GIST: Gastrointestinal stromal tumours
