# Supplementary figures and images for: Diagnosis prediction of tumours of unknown origin using ImmunoGenius, a machine learning-based expert system for immunohistochemistry profile interpretation
Source: Diagn Pathol. 2021 Mar 11;16:19. doi: 10.1186/s13000-021-01081-8 (PMC7953791; doi:10.1186/s13000-021-01081-8)

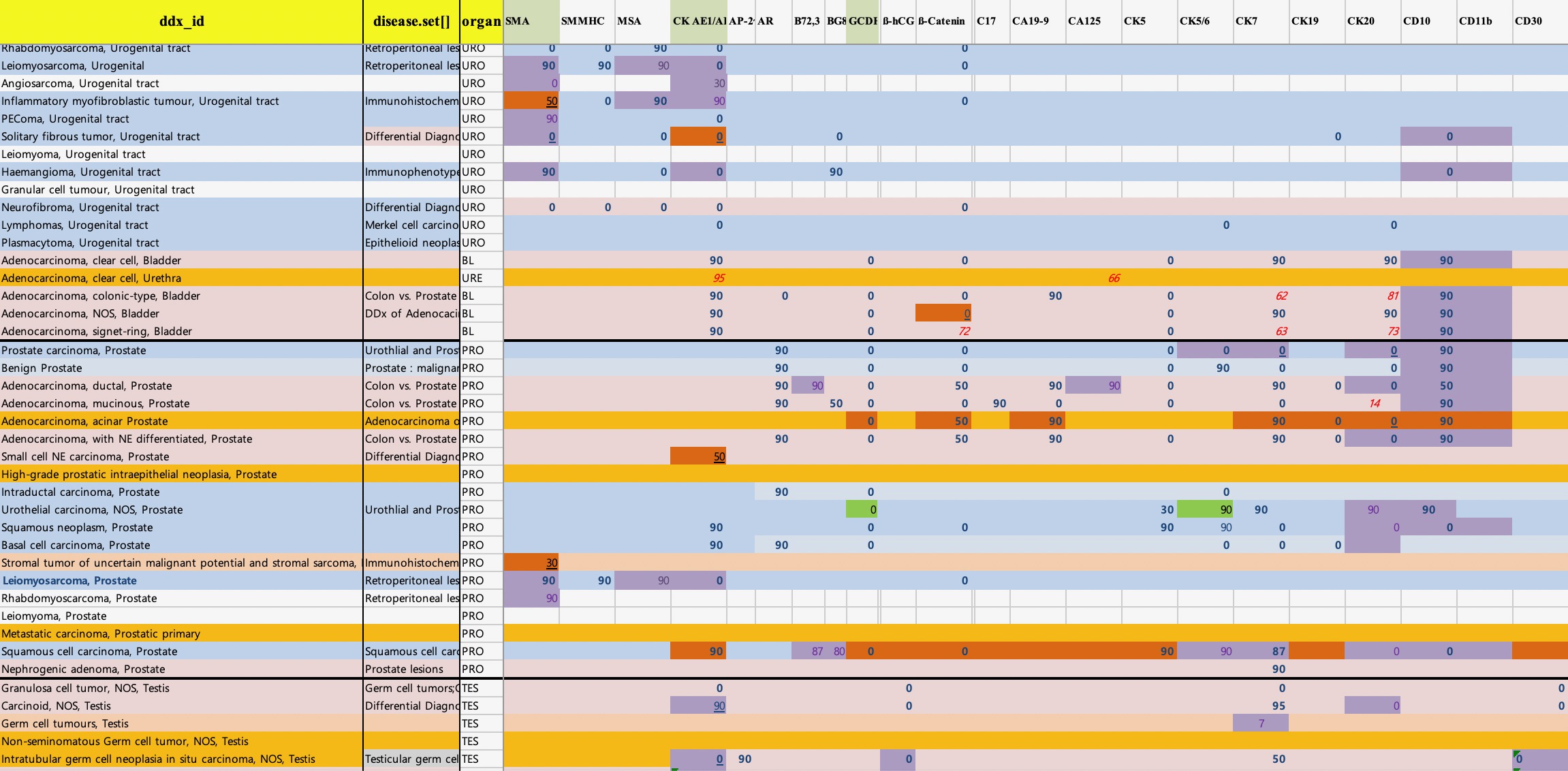

Supplement: Supplementary file 1 — Additional file 1: Figure S1. The example of the IHC database. [file 13000_2021_1081_MOESM1_ESM.jpeg]

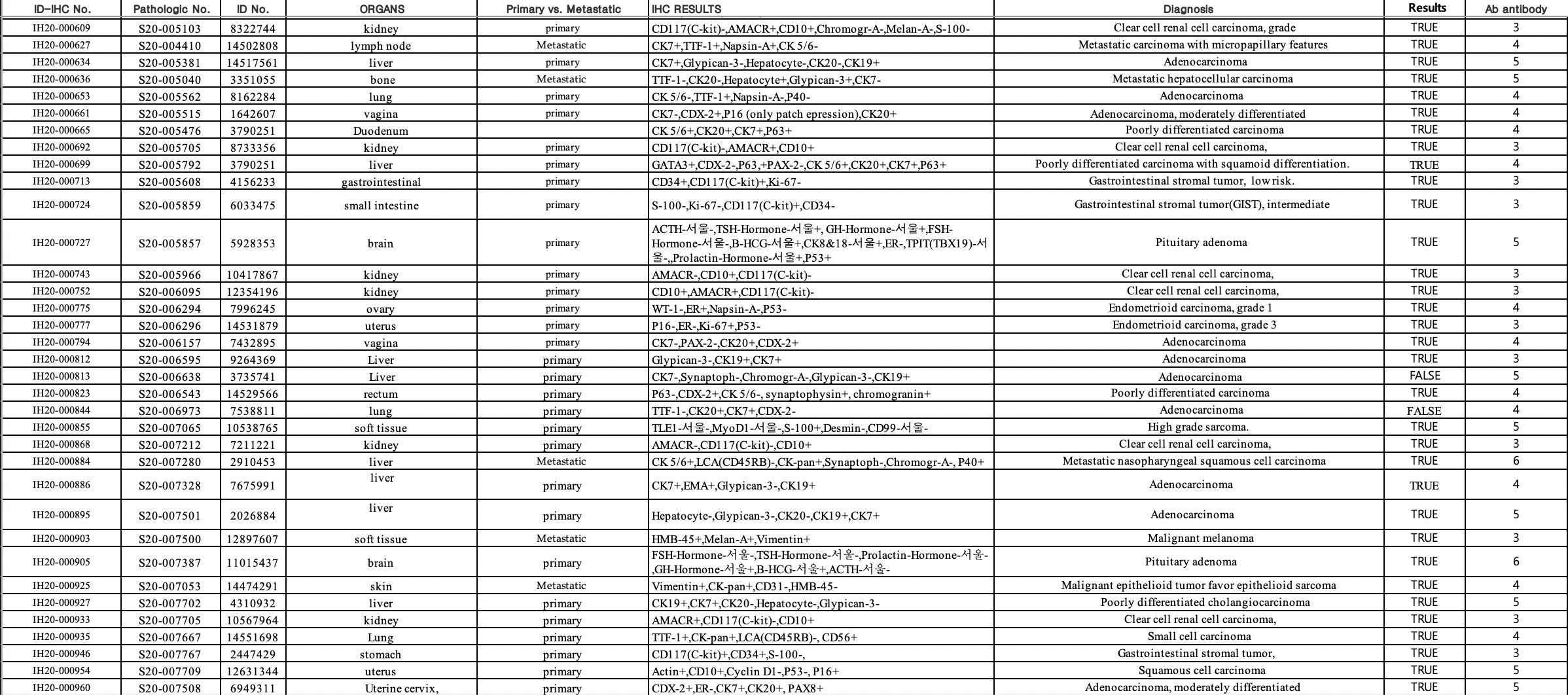

Supplement: Supplementary file 2 — Additional file 2: Figure S2. The example of the patients IHC profile dataset for training and validation of the diagnosis presumption algorithm. [file 13000_2021_1081_MOESM2_ESM.jpeg]

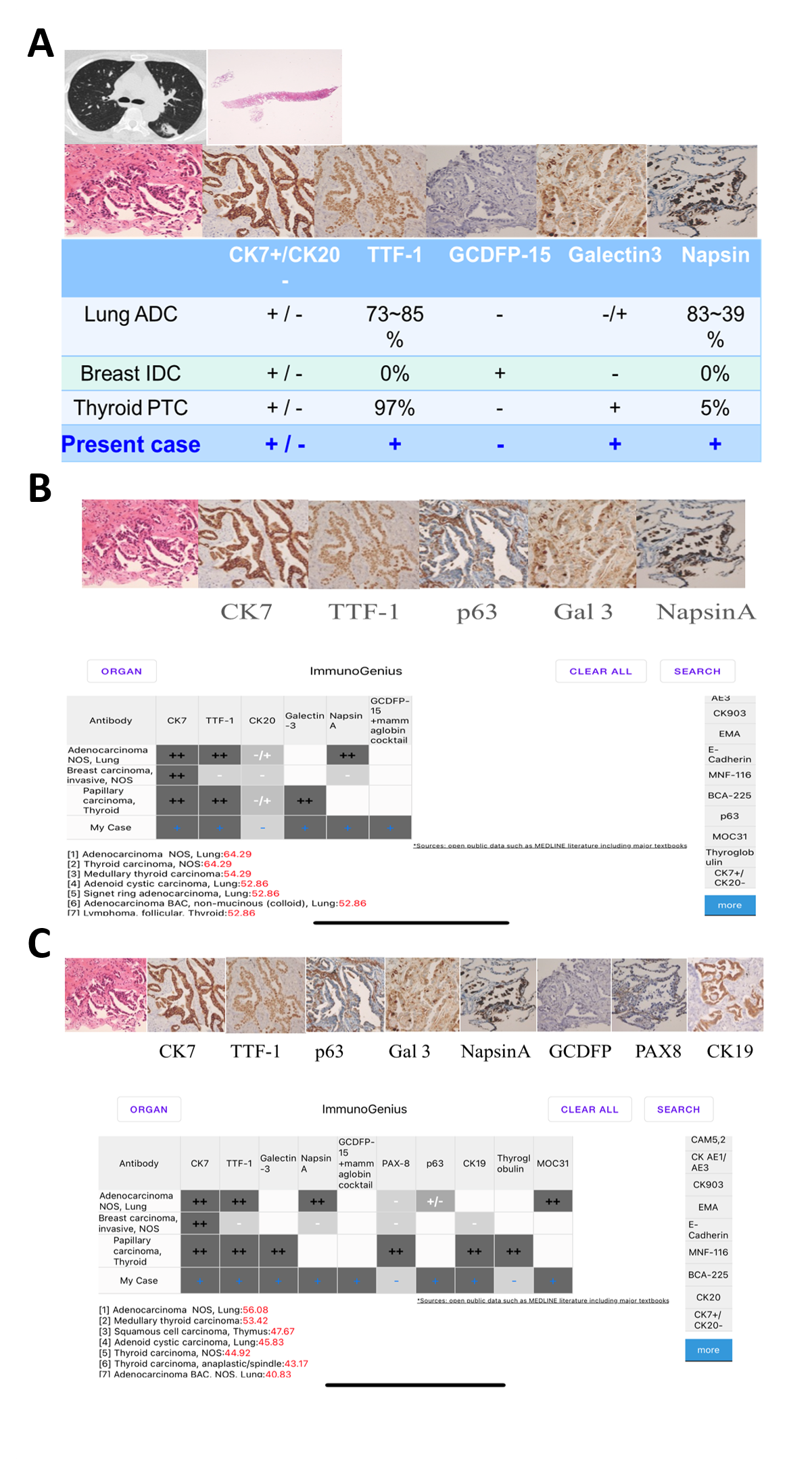

Supplement: Supplementary file 3 — Additional file 3: Figure S3. The example of application using a case of tumor of unknown origin. [file 13000_2021_1081_MOESM3_ESM.tif]
