# Supplementary material for: Diagnosis prediction of tumours of unknown origin using ImmunoGenius, a machine learning-based expert system for immunohistochemistry profile interpretation
Source: Diagn Pathol. 2021 Mar 11;16:19. doi: 10.1186/s13000-021-01081-8 (PMC7953791; doi:10.1186/s13000-021-01081-8)
Supplement: Supplementary file 4 — Additional file 4: Table S1. The reference books used for IHC database build. Table S2. Online references used for IHC antibody name documentation. [file 13000_2021_1081_MOESM4_ESM.docx]

**Table S1.** The reference books used for IHC database build.

| **Title** | **Edition, Year** | **Author, Publisher** |
| --- | --- | --- |
| Diagnostic Immunohistochemistry : Theranostic and Genomic Applications | 4th Edition, 2014 | David J. Dabbs, Elsevier Saunders, Philadelphia, USA |
| Pathology and Geneatics of Head and Neck Tumours 2017 | WHO/IARC Classification of Tumours, 4th Edition, Volume 9, 2017 | IARC, Lyon, France |
| WHO Classification of Skin Tumours 2018 | WHO/IARC Classification of Tumours, 4th Edition, Volume 11, 2018 | IARC, Lyon, France |
| Pathology and Genetics of Tumours of the Lung, Pleura, Thymus and Heart 2015 | WHO/IARC Classification of Tumours, 4th Edition, Volume 10, 2015 | IARC, Lyon, France |
| Pathology and Genetics of Tumours of the Digestive System 2010 | WHO/IARC Classification of Tumours, 4th Edition, Volume 2, 2010 | IARC, Lyon, France |
| WHO Classification of Tumours of Soft Tissue and Bone 2013 | WHO/IARC Classification of Tumours, 4th Edition, Volume 5, 2013 | IARC, Lyon, France |
| WHO Classification of Tumours of Urinary System and Male Genital Organs 2016 | WHO/IARC Classification of Tumours, 4th Edition, Volume 8, 2016 | IARC, Lyon, France |
| Pathology and Genetics of Tumours of Endocrine Organs 2017 | WHO/IARC Classification of Tumours, 4th Edition, Volume 8, 2017 | IARC, Lyon, France |
| WHO Classification of Tumours of the Central Nervous System 2016 | WHO/IARC Classification of Tumours, 4th Edition Revised, Volume 1, 2016 | IARC, Lyon, France |
| WHO Classification of Tumours of the Breast 2012 | WHO/IARC Classification of Tumours, 4th Edition, Volume 6, 2012 | IARC, Lyon, France |
| WHO Classification of Tumours of Haematopoietic and Lymphoid Tissues 2017 | WHO/IARC Classification of Tumours, Revised 4th Edition, Volume 4, 2017 | IARC, Lyon, France |
| Quick Reference Handbook for Surgical Pathologists | 2011 | Rekhtman, Natasha, Bishop, Justin A, Springer, Germany |
| Cell Marque Immunohistochemistry | Reference guide, Vol.11 | Sigma-Aldrich Co., Germany |
| Modern Immunohistochemistry | 2nd Edition, 2014 | Cambridge University Press, United Kingdom |

**Table S2.** Online references used for IHC antibody name documentation.

| Online references | Online address | Version |
| --- | --- | --- |
| Human pathology | http://www.humpath.com | 1996 - 2017 |
| An immunohistochemical vade mecum | http://www.e-immunohistochemistry.info | October 2012 |
| Pathology Outline | http://www.pathologyoutlines.com | 2002 - 2017 |
